# Roles of long noncoding RNA in triple‐negative breast cancer

**DOI:** 10.1002/cam4.6600

**Published:** 2023-10-05

**Authors:** Plabon Kumar Das, Ayesha Siddika, Khan Mohammad Rashel, Abdul Auwal, Kazi Soha, Md. Arifur Rahman, Suja Pillai, Farhadul Islam

**Affiliations:** ^1^ Department of Biochemistry & Molecular Biology Rajshahi University Rajshahi Bangladesh; ^2^ Institute for Glycomics Griffith University Gold Coast Australia; ^3^ Institute of Tissue Banking & Biomaterial Research, Atomic Energy Research Establishment (AERE) Savar Dhaka Bangladesh; ^4^ School of Biomedical Sciences University of Queensland Saint Lucia Australia

**Keywords:** chemotherapy resistance, long noncoding RNAs, pathogenesis, prognosis, radiotherapy resistance, targeted therapies

## Abstract

**Introduction:**

Long noncoding RNAs (lncRNAs) play crucial roles in regulating various hallmarks in cancers. Triple‐negative (Estrogen receptor, ER; Human epidermal growth factor receptor 2, HER2; Progesterone receptor, PR) breast cancer (TNBC) is the most aggressive form of breast cancers with a poor prognosis and no available molecular targeted therapy.

**Methods:**

We reviewed the current literature on the roles of lncRNAs in the pathogenesis, therapy resistance, and prognosis of patients with TBNC.

**Results:**

LncRNAs are associated with TNBC pathogenesis, therapy resistance, and prognosis. For example, lncRNAs such as small nucleolar RNA host gene 12 (SNHG12), highly upregulated in liver cancer (HULC) HOX transcript antisense intergenic RNA (HOTAIR), lincRNA‐regulator of reprogramming (LincRNA‐ROR), etc., are aberrantly expressed in TNBC and are involved in the pathogenesis of the disease. LncRNAs act as a decoy, scaffold, or sponge to regulate the expression of genes, miRNAs, and transcription factors associated with pathogenesis and progression of TNBC. Moreover, lncRNAs such as ferritin heavy chain 1 pseudogene 3 (FTH1P3), BMP/OP‐responsive gene (BORG) contributes to the therapy resistance property of TNBC through activating ABCB1 (ATP‐binding cassette subfamily B member 1) drug efflux pumps by increasing DNA repair capacity or by inducing signaling pathway involved in therapeutic resistance.

**Conclusion:**

In this review, we outline the functions of various lncRNAs along with their molecular mechanisms involved in the pathogenesis, therapeutic resistance of TBNC. Also, the prognostic implications of lncRNAs in patients with TNBC is illustrated. Moreover, potential strategies targeting lncRNAs against highly aggressive TNBC is discussed in this review.

## 1 INTRODUCTION

Globally, triple‐negative breast cancer (TNBC) accounting for approximately 15%–20% of all breast cancers.^1^ It is an aggressive heterogeneous tumor and about 170,000 women are diagnosed with TNBC worldwide every year.^1^, ^2^ Obese women and women aged below 50 years are mostly affected by TNBC.^3^ A moderate five‐year survival rate (77%) is seen for patients with TNBC.^4^

In breast cancers, absence of progesterone receptor (PR), human epidermal growth factor receptor‐2 (HER‐2), and estrogen receptor (ER) in immunohistochemical staining and in situ hybridization known as TNBC.^5^ Generally, TNBC is indicated by massive tumor size, higher tumor grade, presence of lymph nodes metastasis, and poor prognosis along with limited curative options.^6^, ^7^ TNBC patients respond poorly to endocrine and targeted therapies. Combination of surgery, radiotherapy, and cytotoxic chemotherapy regimens, particularly anthracyclines, taxanes, and platinum salts etc., are the preferred approaches in the management of patients with TNBC.^8^, ^9^, ^10^ In spite of that, metastasis and the recurrence rates of TNBC is excessively higher compared to other subtypes of breast cancers.^11^ Also, resistance to conventional therapies could be triggered by several cycles of therapy or intrinsic to the cancers make up.^11^ Therefore, it is of great significance to identify potential therapeutic targets with proven utility as well as clarifying their underlying mechanisms in TNBC tumorigenesis and therapy resistance, which in turn could lead to the development of a new therapeutic strategy for the better management of patients with TNBC.

Long noncoding RNAs (lncRNAs) are a subclass of noncoding RNAs (lncRNAs) that have more than 200 nucleotides and have been implicated in the pathogenesis of various cancers, including TNBC.^12^, ^13^, ^14^, ^15^ It was noted that approximately 80% of the human genome is transcribed to 14,880 lncRNAs from 9277 loci and play crucial roles in the regulation of genes.^13^ They are typical RNA‐type biomolecules transcribed by RNA‐polymerase II (Pol II), harboring a 5′‐methyl‐cytosine cap and a 3′‐poly‐A tail. LncRNAs can be categorized in various groups such as long intergenic ncRNAs (lincRNAs), intronic long ncRNAs (ilncRNAs), promoter‐upstream transcript (PROMPT), promoter‐associated long ncRNAs (paRNAs), repetitive element‐associated long ncRNAs, pseudogene long ncRNAs, enhancer‐associated long ncRNAs etc., based on their size.^15^ They regulate the expression of various genes at different levels, including chromatin, splicing, transcriptional, and post‐transcriptional stages by interacting with their targets. Subsequently, modulation of expression of the target genes involved in vital biological and cellular process such as proliferation, survival, apoptosis, invasion and migration, differentiation, and autophagy in cells, which in turn associated with different pathophysiological conditions, including cancers.^14^, ^15^ Accordingly, they participate in the pathogenesis, progression, and therapeutic resistance of TNBC cells by modulating oncogenic and tumor suppressor pathways. Also, they affect the expression of various miRNAs and transcription factors, thereby regulating the cellular processes.^16^, ^17^, ^18^, ^19^ Aberrant expressions of specific lncRNAs were reported in a broad‐spectrum of breast cancer tissues than in the normal epithelial tissues.^20^ The altered expression of lncRNAs impacted their targets, thereby promoting an aggressive tumor phenotype.^20^ Besides, a number of lncRNAs were downregulated in TNBC tissues and their ectopic overexpression in TNBC cells induced apoptosis and suppressed the proliferation of cancer cells.^21^ In addition, recent studies implied the prognostic significance of lncRNAs in patients with TNBCs.^22^, ^23^, ^24^ Hence, this review aims to extend the current understanding of the roles of lncRNAs in TNBC prognosis, pathogenesis, and therapeutic resistance, therefore, could sheds lights for novel strategies development for patients with TNBCs.

## 2 ROLES OF lncRNAs IN TBNC PATHOGENESIS

**FIGURE 1:**
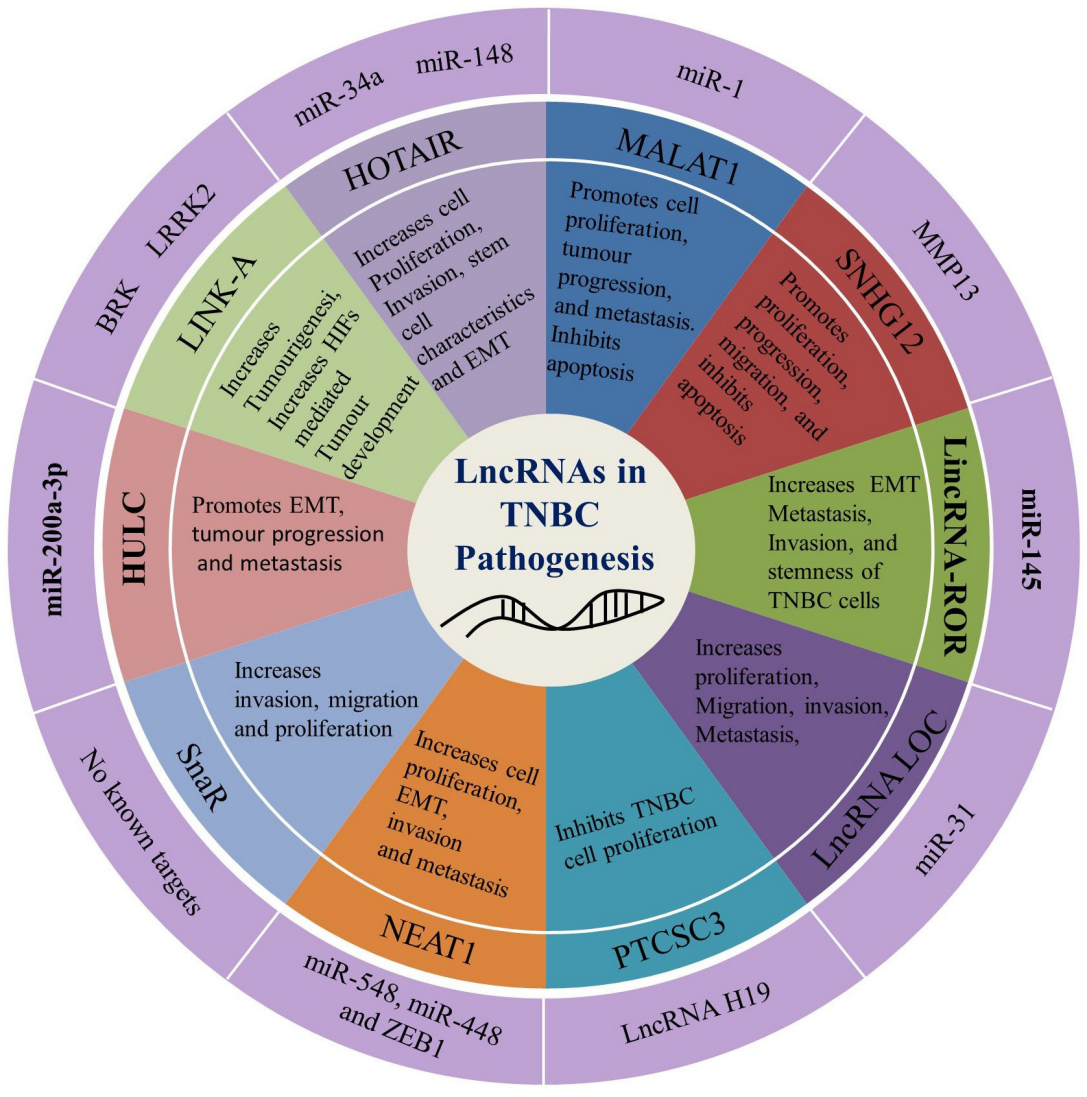
Functions of long noncoding RNAs in TNBC. LncRNAs positively or negatively regulates the proliferation, invasion, metastasis, and stemness property of TNBC cells by regulating the expression of miRNAs, or transcription factors. The outer circle presents the miRs targets, while the second circle showing the lncRNAs. The next circle shows the biological and cellular processes regulated by the lncRNAs in TBNC.

The definite functions of lncRNAs in cancer pathogenesis is yet to be unveiled; however, in recent years researchers noted altered expression of lncRNAs in TNBCs, indicating their roles in the pathogenesis of TNBC (Figure 1). For example, a number of lncRNAs could act as having oncogenic potential, that is, promoting TNBC, whereas other types of lncRNAs suppressed TNBC pathogenesis and progression.^25^, ^26^, ^27^ A list of involved lncRNAs along with their potential functions in TBNC pathogenesis are summarized in Table 1.

**TABLE 1:** Roles of lncRNAs in the pathogenesis of TNBC.

LncRNAs	Expression pattern	Targets	Molecular functions	Reference
HOTAIR	Upregulated	miR‐34a miR‐148	Increases metastasis of TNBC cells by inhibiting the expression of metastasis‐suppression genes Inhibits miR‐34a expression to release its inhibitory effects towards stemness associated genes *Sox‐2* Increases the proliferation of TNBC cells by binding with promoters of tumor suppressor p53 and p21. HOTAIR can also indirectly suppress miRNA‐148 expression in TNBC and increased cell invasion and metastasis	30, 31
MALAT1	Upregulated	miR‐1	Promotes cell proliferation, tumor progression, metastasis, and inhibits apoptosis of TNBC cells both in vivo and in vitro	^35^
LINK‐A/LOC339535	Upregulated	BRK and LRRK2	Promote tumourigenesis by recruiting breast tumor kinase (BRK) activated together with leucine‐rich repeat kinase 2 (LRRK2) which increase HIF1α driven proliferation of TNBC cells	^40^
SNHG12	Upregulated	MMP13	Promotes proliferation, progression, migration, and inhibits apoptosis	41, 42, 43
LincRNA‐ROR	Upregulated	miR‐145	Control the expression of various stemness factors including SOX2, OCT4, and NANOG, by sponging the effect of miR‐145 Increases EMT, metastasis, invasion, and stemness of TNBC cells	47, 49
HULC	Upregulated	miR‐200a‐3p	Increases EMT, tumor progression, and metastasis	^53^
SnaR	Upregulated	No known targets	Increases invasion, migration, and proliferation	^57^
NEAT1	Upregulated	miR‐448 miR‐548 and ZEB1	Regulates apoptosis and cell cycle progression. NEAT1 is also involved in chemotherapy resistance as knockdown of NEAT1 sensitized cells to chemotherapy treatment	59, 60, 61
PTCSC3	Downregulated	LncRNA H19	Suppresses TNBC cell proliferation by downregulating lncRNA H19	^62^
LOC (LOC554202)	Upregulated	miR‐31	Increases tumourigenesis, invasion, migration, and inhibits apoptosis	^63^

LncRNA HOX transcript antisense intergenic RNA (HOTAIR) is one of the first lncRNA identified in breast cancer.^28^ It acts as a scaffold to assemble epigenetic moderators to regulate gene expression.^29^ HOTAIR is overexpressed in patients with major breast cancer subtypes including in TNBC. HOTAIR aggravates cancer metastasis by inhibiting the expression of metastasis‐suppression miRNAs such as miR‐148 and miR‐34a.^30^, ^31^ It promotes the malignancy of TNBC through a variety of ways like increasing invasion, metastasis, and stemness of breast cancer cells.^30^ For example, its pro‐oncogenic activity is mediated in part by its interaction with the polycomb repressive complex 2 (PRC2).^30^ Also, HOTAIR acts as a miR‐34a sponge, where it releases the inhibitory effects of miR‐34a towards its stemness associated target gene SOX‐2.^31^ On top of that HOTAIR increases the proliferation of MDA‐MB‐231 TNBC cells by binding with promoters of tumor suppressors p53 and p21. Furthermore, a negative correlation was noted between HOTAIR and miR‐148. It induces downregulation of miRNA‐148 expression indirectly, which in turn increased invasion and metastasis of cells. Also, increase the breast cancer stem cell population, and enhanced epithelial–mesenchymal transition (EMT) partially.^30^ Thus, inhibition of HOTAIR expression in TNBC could be a potential option as far as RNA‐based therapy of TNBC is concerned. Interestingly, combination of lapatinib with imatinib treatment transcriptionally suppressed HOTAIR expression in TNBC cells through inhibition of β‐catenin‐binding sites of lymphoid enhancer‐binding factor 1 LEF1/TCF4 (Transcription factor 4), which in turn causes inhibited MDA‐MB‐231 TNBC cell's growth.^32^ Also, treatment of cancer (MDA‐MB‐231) cells derived from TNBC with phenolic compound such as Delphinidin‐3‐glucoside could halt HOTAIR expression both in vivo and in vitro.^33^ This information elucidated mechanisms, which were previously unidentified in TNBC pathogenesis, thus, could offers a new‐horizon for developing therapies for patients with TNBC targeting HOTAIR expression.

Another highly conserved lncRNA, metastasis associated lung adenocarcinoma transcript 1 (MALAT1) regulates the expression of genes via modulating transcription and post‐transcriptional RNA processing in various cancers.^34^, ^35^, ^36^, ^37^, ^38^ MALAT1 was first reported in non‐small cell lung cancer (NSCLC) and its expression was associated with metastasis and poor survival of patients.^34^ Later, it was reported that MALAT1 promotes the progression of TNBC by inhibition of apoptosis of cancer cells along with stimulating metastasis followed by cell proliferation.^38^, ^39^, ^40^, ^41^, ^42^, ^43^, ^44^, ^45^ MALAT1promotes TNBC through interacting with microRNA‐1 (miR‐1), downregulation of MALAT1 increased the expression of miR‐1, while overexpression of miR‐1 decreased MALAT1 expression in TNBC.^35^ In TBNC, the activities of MALAT1 interacting protein partners and its target genes are potentially unique such as other subtypes of breast cancers. Importantly, overexpression of MALAT1 inhibited apoptosis, whereas suppression of MALAT1 promoted apoptosis cells derived from TBNC. Thus, targeting MALAT1 could induce apoptosis of TNBC cells. Treatment of cancer cells derived from TBNC with high concentration of 17β‐estradiol (E2) induce reduction in MALAT1 mRNA expression by post‐transcriptional degradation,^39^ which indicates targeting the expression of MALAT1 in TNBC could be useful option for therapy development.

A cytoplasmic lncRNA with prognostic significance called long intergenic noncoding RNA for kinase activation (LINK‐A) is identified in TNBC.^40^ A significant higher expression of LINK‐A was noted in stage‐III TNBC tissues compared to non‐neoplastic adjacent breast tissues, and tissues obtained from other subset of breast cancers (ERPR+/HER2+, HER2‐/ERPR+, AND ERPR‐/HER2+). LINK‐A plays critical role in growth factor‐mediated normoxic hypoxia‐inducible factor 1‐alpha (HIF1α) signal transduction pathway.^40^ LINK‐A expression and LINK‐A‐dependent signaling pathway activation associated with TNBC progression and LINK‐A overexpressed patients had poorer progression‐free survivals.^40^ TBNC pathogenesis can be promoted by switching the LINK‐A dependent signaling pathways followed by breast tumor kinase recruitment along with activated leucine‐rich repeat kinase 2 (LRRK2). LRRK2 phosphorylates HIF1α and this phosphorylation prevents HIF1α degradation under normoxic conditions. Phosphorylation of HIF1α leads to activation of HIF1α target genes upon heparin‐binding EGF‐like growth factor (HB‐EGF) stimulation, which subsequently promotes TNBC tumorigenesis. As HIF1α signaling pathway is very crucial in TNBC development, therefore, both LINK‐A expression and LINK‐A‐mediated activation of normoxic HIF1α signaling pathway could be a potential target against TNBC.^40^ However, a deep understanding, especially whether LINK‐As are released into circulation continuously via the apoptosis of cancer cell or actively secreted from TNBC cells is yet to be established.

Upregulation of small nucleolar RNA host gene 12 (SNHG12) in TNBC, promotes cellular proliferation, migration, and progression, and inhibits the programmed cell dead apoptosis. SNHG12 is also upregulated in some other cancers, including nasopharyngeal carcinomas, endometrial carcinomas, and osteosarcomas.^41^, ^42^ A study reported significant upregulation of SNHG12 expression in TNBC tissues (*n* = 102) in comparison to that of noncancerous breast (*n* = 95) tissues.^43^ Moreover, presence of lymph node metastasis and larger tumor size were statistically linked to SNHG12 overexpression (*p* < 0.05). Mechanistically, in TBNC, transcription factor c‐MYC targets the expression of SNHG12 directly and induces overexpression of SNHG12, thereby promoting the proliferation and migration of cells derived from TNBC (BT‐549 and MDA‐MB‐231).^43^ Also, siRNA‐mediated suppression of c‐MYC induced reduction in SNHG12‐regulated effects in TNBC cells. Moreover, proliferation and induction of apoptosis of TNBC cells is inhibited by silencing of SNHG12. Furthermore, SNHG12 regulates the expression of matrix metalloproteinase 13 (MMP13), thereby promoting the migration of cells.^43^ The expression of SNHG12 is positively correlated with the expression of MMP13, thus, MMP13 induced degradation of extracellular matrix in TNBC cells promotes tumor invasion and metastasis. Also, MMP13 is stabilized by SNHG12 via acting as a scaffold‐mediating RNA‐binding protein or a competing endogenous RNA (ceRNA).^43^ However, further studies including in vivo experiments are still required to properly understand the biological effects of SNHG12, especially in proliferation and apoptosis mediated by SNHG12 in cells derived from TBNC.

Long intergenic noncoding RNA‐regulator of reprogramming (lincRNA‐ROR) is another important lncRNA involved in the regulation of reprogramming process in embryonic stem and differentiated cells.^44^ The lincRNA‐ROR is highly overexpressed in TNBC tissues when compared to that of noncancerous tissues.^45^ It could promote cancer pathogenesis, especially metastasis via regulating the epithelial to mesenchymal transition (EMT), whereas silencing of lincRNA‐ROR suppressed EMT phenotype in TNBC cells.^45^ Deep sequencing analysis revealed that miR‐145 downregulation is a hallmark of metastasis, which is regulated by lincRNA‐ROR.^46^ LincRNA‐ROR might serve as a ceRNA against miR‐145 to limit the expression of miR‐145 in TNBC.^47^ Reportedly, both miR‐145 and lincRNA‐ROR have been associated with various processes of embryonic and adult stem cells development.^48^ Also, lincRNA‐ROR regulates the expression of various stemness factors, including SOX2, OCT4, and NANOG by sponging the effect of miR‐145.^49^ In TNBC, lincRNA‐ROR dramatically upregulated which results in miR‐145 downregulation and miR‐145 expression restored by suppressing lincRNA‐ROR expression.^47^ It is noted that miR‐145 regulates TBNC cells invasion by regulating the expression of GTPase ADP‐ribosylation factor 6 (ARF6), a novel target and crucial mediator of invasion of breast cancer cells.^47^ ARF6 mRNA was degraded by miR‐145 as it directly binds to 30′UTR, thereby inhibits its expression. Thus, lincRNA‐ROR inhibits miR‐145 expression, which leads to Arf6 mediated invasion of TNBC cells.^47^ Therefore, in TBNC the lincRNA‐ROR/miR‐145/ARF6 signaling axis regulates the invasion and metastasis cancer cells.

Highly upregulated in liver cancer (HULC), another lncRNA overexpressed in a variety of human cancers including TNBC.^50^, ^51^, ^52^, ^53^ HULC acts as an oncogene in the development and progression of tumor.^52^ HULC mediates its function by modulating miR‐200a‐3p/ZEB1 signaling pathway, which in turn promotes EMT, thereby leads to tumor metastasis.^53^ Overexpression of HULC associated with the proliferation, invasion, migration, metastasis, and adverse prognosis of TBNC patients.^50^ Also, in vitro invasion and migration of TBNC cells inhibited followed by silencing of HULC expression.^50^ HULC drives TNBC cells migration and invasion by inducing overexpression of MMP‐2 and MMP‐9 in MDA‐MB‐231 and BT549 cells.^50^ However, expressions of genes associated with EMT such *E‐cadherin*, *vimentin*, *Snail*, and *Slug* were not altered by suppressing HULC in MDA‐MB‐231 and BT549 cells, which further confirmed MMP‐2 and MMP‐9 mediated invasion activity of HULC in TNBC cells.^50^ Though, the inhibition of HULC expression did not affect on TNBC cells growth and proliferation; however, prohibited invasion, migration, and metastasis of TBNC cells, thus, it has the potential to be used as therapeutic target.

Small NF90‐associated RNA (SnaR) is a double‐stranded lncRNA, involved in cancer cell growth.^54^, ^55^, ^56^ SnaR predominantly upregulated in TNBC (MDA‐MB‐231) cells when compared to that of non‐TBNC (MCF7) breast cancer cells.^57^ Silencing of SnaR by siRNA induce reduction in proliferation, invasion, and migration of cells (MDA‐MB‐231) derived from TNBC significantly.^57^ Though, suppression of snaR could restrict proliferation, invasion, and migration of cells derived from TNBC, the underlying mechanism requires to be explored in further studies.

Also, in TBNC the expression of lncRNA nuclear enriched abundant transcript 1 (NEAT1) is overexpressed significantly.^58^ It promotes cancer progression by stimulating cell proliferation, EMT, invasion, and metastasis.^58^ Also, NEAT1 promotes TNBC cells growth by regulating apoptosis and cell cycle progression. Furthermore, NEAT1 induced chemotherapy resistance in TNBC cells, whereas knockdown of NEAT1 sensitized TNBC cells to chemotherapy.^59^ Mechanistically, it promoted breast cancer growth by regulating miRNAs such as miR‐548 and miR‐448 and ZEB1.^60^, ^61^ Over expression of NEAT1 inhibits the expression of miR‐448, thereby freeing ZEB1 to mediate its action. Besides, suppression of NEAT1 resulted in decreased CD44^high^, CD24^low^, ALDH^high^, and SOX2^high^ cancer stem cells populations, a population with self‐renewal and multilineage differentiation properties.^60^ Also, NEAT1 overexpression is associated with poor survival rates of patients. Thus, considering the roles of NEAT1 in cancer promotion, chemoresistance, and cancer stemness, it was suggested that it could be used as a new clinical therapeutic target for treating TNBC patients.

LncRNAs can also inhibit the function of another lncRNAs, for example, lncRNA PTCSC3 suppresses the expression of lncRNA H19, resulting in inhibition of cells proliferation derived from TBNC.^62^ PTCSC3 was downregulated in TNBC whereas H19 was upregulated. PTCSC3 inhibits the proliferation of TNBC cells, whereas cell migration and invasion were not significantly affected by PTCSC3 overexpression.^62^ LOC554202, another lncRNAs overexpressed in breast cancer tissues and cells (MDA‐MB‐231) derived from TBNC, thereby promotes tumorigenesis.^63^ Silencing of LOC554202 diminished cancer cell proliferation, increased apoptosis, and inhibited migration/invasion in vitro and halted tumorigenesis in vivo by regulating miR‐31 in TNBC cells.^63^

## 3 ROLES OF lncRNA IN TNBC CHEMOTHERAPY RESISTANCE

**FIGURE 2:**
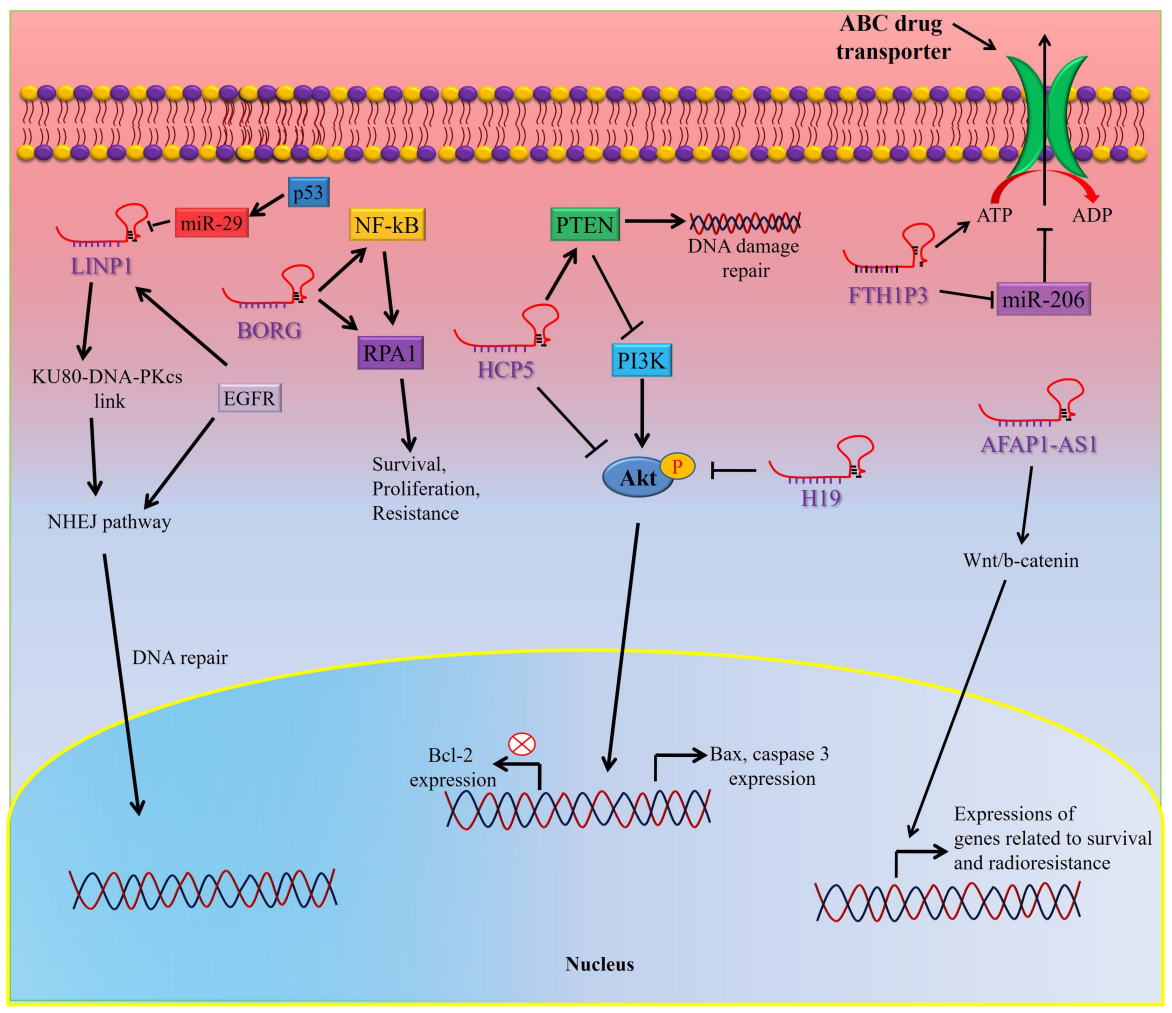
Role of lncRNAs in chemotherapy resistance of TNBC. LncRNAs regulates chemotherapy resistance by inducing the activation of growth signaling pathways, increasing the expression of drug efflux pumps, promoting DNA damage repair, or by inhibiting apoptosis.

Therapy resistance is a major limitation in the treatment of patients with TNBC. Both the acquired (due to prolonged drugs use) and intrinsic (pre‐existing) therapy resistance can develop in patients with TNBC.^64^ Dysregulation of lncRNAs is associated with critical functions in regulating chemoresistance in TNBC.^65^, ^66^ Though they are implicated in both inhibition and promotion of chemoresistance, by acting as ceRNA most of them stimulate chemoresistance in TNBC, thereby sponging miRNAs functionality in TNBC (Table 2). LncRNAs target drug efflux pump of ATP‐binding cassette (ABC) transporter superfamily or and mediate MDR (multidrug resistance) in various cancers, including TNBC (Figure 2).^67^ For example, lncRNA FTH1P3 (ferritin heavy chain 1 pseudogene 3) activates taxol drugs (paclitaxel) resistance in TNBC through modulating miR‐206/ABCB1 axis.^68^ The most canonical chemoresistance protein ABCB1 is expressed in multidrug resistant cancer.^69^, ^70^ The expression of lncRNA FTH1P3 upregulated in MDA‐MB‐231/PTX, a paclitaxel‐resistant cells in comparison to paclitaxel‐sensitive cells and silencing of FTH1P3 increased the sensitivity of paclitaxel (50%) treatment by arresting cells at G2/M phase.^68^ Also, reduced ABCB1 protein expression and tumor growth was noted in mouse xenotransplanted model using paclitaxel‐resistant cells derived from TBNC followed by FTH1P3 silencing. Mechanistically, FTH1P3 interacts with miR‐206 and regulate its targets expression. In addition, miR‐206 targets the 3′‐UTR of mRNA encoding ABCB1, thus, upregulation of FTH1P3 and ABCB1 in paclitaxel‐resistant cancer cells derived from TBNC suggested FTH1P3 targets miR‐206, thereby freeing ABCB1 expression. Thus, lncRNA FTH1P3 plays important functions in paclitaxel resistance in TNBC via regulating the miR‐206/ABCB1 signaling axis, unveiling a novel molecular insight regarding TNBC chemotherapy resistance.^68^

**TABLE 2:** LncRNAs associated with chemoradiotherapy resistance in TNBC.

LncRNAs	Expression pattern	Target (s)	Molecular functions	Reference
FTH1P3	Upregulated	miR‐206/ABCB1	Promotes ABCB1 protein expression by sponging miR‐206 thereby activating subsequently paclitaxel resistance in TNBC cells	^68^
BORG	Upregulated	RPA1	Enhances the survival of TNBC cells and allows them to become resistant against doxorubicin by activating RPA1 expression	^72^
H19	Upregulated	Akt signaling	Increases chemoresistance to paclitaxel by inhibiting the expression of Bax, caspase 3 and enhancing the expression of Bcl‐2	^77^
HCP5	Downregulated	PTEN	Reduces cisplatin resistance of TNBC cells Upregulates the expression of PTEN and downregulate p‐Akt expression	^79^
lncAFAP1‐AS1	Upregulated	Wnt signaling	Activates Wnt/β‐catenin signaling to promote radioresistance in TNBC cells by inducing cell proliferation, migration, and invasion.	^2^
LINP1	Upregulated	No known targets	Increases double‐strand DNA break (DSB) repair by serving as a scaffold that links Ku80 with DNA‐PKcs Increases NHEJ pathway mediated radioresistance in TNBC cells	^82^

BMP/OP‐responsive gene (BORG), a prometastatic lncRNA is overexpressed in TNBC cells in stressed environment during metastasis.^71^, ^72^ This stress‐induced expression of BORG enhances the survival of TNBC cells and more importantly allows them to become resistant against chemotherapeutic agent doxorubicin both in vitro and in vivo.^72^ However, this BORG‐dependent chemoresistant trait of TNBC cells largely depends upon the activation of the NF‐κB signaling axis.^72^ NF‐κB is a vital signaling pathway which plays critical role in breast cancer to acquire chemoresistant phenotype and this signaling pathway are often hijacked by malignant cells such as TNBC cells,^73^, ^74^ particularly after therapeutic intervention.^75^, ^76^ BORG mediates its action via a novel feed‐forward signaling loop, by binding and activating replication protein A1 (RPA1). NF‐κB signaling pathway inhibition or prevention of DNA‐binding activity of RPA1 by genomic and pharmacologic intervention decreased prosurvival features of BORG.^72^ On top of that, abrogation of BORG activity makes TNBCs sensitive to doxorubicin‐induced cytotoxicity. Therefore, therapeutic intervention of BORG expression or its downstream target could provide a novel means to minimize TNBC therapy resistance.

LncRNA H19 is another crucial RNA overexpressed in 70% breast cancer and involved in the regulation of chemoresistance.^77^ LncRNA H19 induced chemoresistance in TNBC cells and highly overexpressed in paclitaxel‐resistant TNBC cells compared to paclitaxel‐sensitive cells. Whereas knockdown of H19 restored paclitaxel resistance of TNBC cells by triggering Akt mediated apoptosis.^77^ Aberrant activation of Akt signaling is quite a common phenomenon in breast cancer therapy resistance. Indeed, inhibition of Akt signaling by Ipatasertib combined with paclitaxel treatment enhanced the median progression‐free survival in comparison to lone paclitaxel treatment in metastatic TNBC patients.^78^ Thus, Akt‐targeted therapy should be a potential strategy for TNBC treatment.^78^ Overexpression of H19 decreases the phosphorylation of Akt, which subsequently increases the expression of its downstream targets such as *Bax* and *caspase 3* and decreases the expression of apoptotic *Bcl‐2* gene.^77^ Therefore, H19 could be an effective therapeutic option in paclitaxel‐resistant TNBC cells.

Additionally, histocompatibility leukocyte antigen complex P5 (HCP5) is a lncRNA which is significantly downregulated in cisplatin resistant TNBC (MDA‐MB‐231/DDP) cells in comparison to cisplatin sensitive MDA‐MB‐231 cells.^79^ Downregulation of HCP5 in TNBC cells associated with cisplatin (DDP) resistance; however, overexpression of HCP5 resulted in increased sensitivity against therapy in DDP‐resistant TNBC cells both in vitro and in vivo. Overexpression of HCP5 upregulates the expression of phosphatase and tensin homolog (PTEN) and downregulates the expression of p‐Akt in TNBC cells.^79^ PTEN involved in therapy resistance against DNA damaging drugs in cancers by enhancing DNA repair capacity of cancer cells.^80^, ^81^ Thus, downregulation of HCP5 promoted DDP resistance by regulating the expression of PTEN and p‐Akt in TNBC.

## 4 ROLES OF lncRNAs IN TNBC RADIOTHERAPY RESISTANCE

Resistance to radiotherapy leads to enhanced local invasion, metastasis, and poor prognosis of cancer patients, which is a common problem in clinics and contributed worst clinical outcome.^1^ LncRNAs play important roles in the development of radioresistance by regulating the expression of target genes associated with radioresistance (Table 2). Exploring key lncRNAs along with their mechanisms attributed radioresistance would be beneficial to develop effective therapeutic modalities, which, however, remains challenging and could minimize radioresistance in patients with cancers. Recently, a number of radioresistance‐associated lncRNAs such as actin filament‐associated protein 1 antisense RNA1 (lncAFAP1‐AS1), lncRNA in nonhomologous end‐joining pathway 1 (LINP1) have been identified in TNBC.^2^ The lncRNA, lncAFAP1‐AS1 induced radioresistance through triggering the canonical Wnt/β‐catenin signaling pathway in cells derived from TNBC.^2^ Also, overexpression of LncAFAP1‐AS1 was noted in radioresistant patients with TNBC, while it promotes cell proliferation, invasion, and migration via activating Wnt/β‐catenin signaling pathway.^2^ Silencing of lncAFAP1‐AS1 resulted in improved radiosensitivity in TNBC cells followed by reduction in reactive oxygen species‐mediated radioresistance both in vivo and in vitro.

LINP1 is an intergenic lncRNA, which plays crucial role in promoting TNBC cell proliferation, progression, metastasis, and radioresistance.^82^ It overexpressed in TNBC in comparison to other subtypes of breast cancers.^82^ Also, LINP1 increases double‐strand DNA break (DSB) repair by serving as a scaffold that links Ku80 with DNA‐PKcs.^82^ The link between Ku80 and DNA‐PKcs activates nonhomologous end‐joining (NHEJ) pathway, which is a major pathway in tumor cells that respond to radiation treatment.^83^, ^84^, ^85^, ^86^, ^87^ Inhibition of this pathway in combination with DNA‐damaging therapies has been implicated in TNBC.^88^, ^89^ Though LINP1 expression is not essential for NHEJ activity; however, LINP1 expression caused increases activity of NHEJ repair pathways. Furthermore, epithelial growth factor receptor (EGFR) pathway promotes DNA repair capacity by NHEJ pathway and EGFR was reported to be highly overexpressed in TNBC. Another mechanism was proposed as activation of EGFR results in upregulation of *LINP1* transcription through RAS–MEK–ERK pathway and activation of AP1 transcription factors.^82^ Therefore, EGFR activation increases LINP1 level, which stabilizes DNA‐PKcs and Ku80 interaction, thus, stimulates DNA repair activity via NHEJ‐mediated pathway. On the contrary, activation of p53 decreases LINP1 expression by inducing miR‐29 activation, which targets and inhibits LINP1 RNA. It was noted that LINP1 downregulation by mir29 happened after a long delay, which suggested miR‐29 and p53‐mediated modulation of LINP1 expression could restricts DNA repair activity in cells long after damage via NHEJ‐mediated pathway.^82^ Furthermore, it was further suggested that higher *EGFR* amplification enhances LINP1 expression at the transcriptional level whereas increased *TP53* mutations also promotes post‐transcriptional LINP1 expression in TNBC. Thus, a detailed mechanism of LINP1‐mediated DNA break repair and its regulation could help reducing radioresistance in TNBC cells. However, exploring the sets of lncRNA associated with radioresistance is yet to be established, thus, further researches are imperative to discover the full functional lncRNAs along with their mechanisms involved in TNBC radioresistance.

## 5 PROGNOSTIC IMPLICATIONS OF lncRNAs IN TNBC

Accumulating evidence suggested that expression of lncRNAs could be aberrant in patients with TNBC, thus, they would have the potential to be use patient's prognosis in clinical settings (Table 3). LncRNAs such as HOTAIR, SNHG12, LincRNA‐ROR, HULC etc., are dysregulated in TNBC and their expressions are attributed either negatively or positively in the development and progression of TNBC.^43^, ^47^, ^50^, ^90^, ^91^ Therefore, they have the potential to be used as prognostic tool in TNBC. Interestingly, several individual or combined lncRNAs have been demonstrated to play better prognostic performance than conventional cancer biomarkers. For example, lncRNA MALAT 1 acts as an effective prognostic marker for stage I non‐small cell lung cancer patient.^92^ Furthermore, a meta‐analysis reported the indispensable prognostic implications of lncRNAs in patients with TNBC.^90^ The study vigorously investigated 21 previous studies and noted that 27 lncRNAs have prognostic significance in patients with TNBC. They noted that upregulation of four lncRNAs (MIR503HG, GAS5, TCONS_l2_00002973, and NEF) is associated with increased overall survival of patients with TNBC.^90^ Whereas, elevated expressions of 23 other lncRNAs such as MALAT1, HOTAIR, HIF1A‐AS2, LINC000173, SNHG12, HULC, LINC00096, ZEB2‐AS1, LUCAT1 etc., were associated with poor disease‐free survival of patients with TNBC.^90^ These results suggest potential use of lncRNA as an effective prognostic tool in patients with TNBC.

**TABLE 3:** Prognostic implications of lncRNAs in patients with TNBC.

LncRNAs	Expression	Prognostic value	Reference
HOTAIR	Upregulated	High levels of HOTAIR expression have been linked to poor clinical outcomes and a poor prognosis of patients with breast cancer.	30, 31, 107
MALAT1	Upregulated	Dysregulation of MALAT‐1 expression associated with higher levels of mortality rate (more than 40%) of TBNC patients.	35, 92
LINK‐A/LOC339535	Upregulated	Higher levels of LINK‐A correlated with unfavorable recurrence‐free survival for TBNC patients.	40, 108
SNHG12	Upregulated	A higher expression of SNHG12 associated with worse overall survival and recurrence‐free survival.	41, 42, 43, 109
LincRNA‐ROR	Upregulated	Over expression was linked to shorter overall survival and disease‐free survival in TBNC, as well as chemotherapy resistance. Also, higher lncRNA ROR expression correlated with lymph node metastasis in TBNC patients.	47, 49, 110
HULC	Upregulated	Overexpression is associated with poor survival of patients with TBNCs.	50, 53
NEAT1	Upregulated	High expression of NEAT1 in TNBC patients was linked to a poor overall survival rate.	59, 60, 61

## 6 LncRNAs TARGETED CANCER THERAPY

Recent studies suggest lncRNAs as a target for developing promising therapeutics for various cancers because of their flexible and complex structures and most importantly because of their participation in complex cellular networks. Moreover, selective killing of cancer cells can also be done by targeting specific expression of lncRNAs. In addition, as lncRNAs are expressed in smaller amount, it permits a lower dose of lncRNA targeting drugs, which helps to avoid toxicities of conventional therapies.^93^, ^94^ Considering these, a number of approaches are being developing to inhibit tumor promoting lncRNAs or suppressing their oncogenic effects in cancer progression (Table 4). Moreover, pharmacological intervention to alter their functions have been developing to nullified their cancer stimulating activities.^95^

**TABLE 4:** Novel therapeutic approaches targeting lncRNAs for patients with TBNCs.

Therapeutic strategies	Targeting lncRNA	Possible outcome	Reference
Antisense Oligonucleotides Example, Gapmer	MALAT1 and RPSAP52	One can reduce or change gene expression through steric obstruction, splicing changes, or the start of target degradation.	111, 112, 113
RNAi	HOTAIR	Block lncRNA‐protein interaction	^114^
CRISPR/Cas9	UCA1, lncRNA‐21A, AK013948, and MALAT1	Deleted the target	114, 115
Gene therapy	PTENp1, MEG3, and lncRNA‐p21	Decrease in tumor size because to the high levels of diphtheria toxin produced	114, 116, 117
Small molecule inhibitors Such as PRC2, CBX7, and EXH2	HOTAIR, ANRIL, and H19	Block lncRNA‐protein interaction	^114^
Aptamer	HOTAIR	Potently inhibited the growth, migration, and invasion of EGFR expression	^118^
PNA	HOTAIR	Effectively blocks the HOTAIR‐PRC2 interaction, inhibits ovarian and breast cancers	^119^
Locked nucleic acids GapmeRs	XIST and MALAT1	Resistance to enzymatic degradation. RNase‐H‐mediated destruction, effective interaction with the secondary structure of lncRNAs, or inhibiting the translational machinery	52, 95
Natural Molecules e.g., resveratrol and curcumin	NEAT1, H19, and MALAT1	Through NEAT1/Wnt/−catenin, resveratrol prevents MM cells from proliferating and migrating. H19 can be downregulated by curcumin, which can also increase the expression of p53.	^120^

Oncogenic lncRNAs are generally overexpressed in patients with cancers, thus, inhibitors or antagonist can be designed to target them, thereby reduced their level of expression in cancers. Those techniques or methods include using antisense oligonucleotides (ASOs), small interfering RNAs (siRNA), using aptemers etc., which could inhibit the expression of lncRNAs or hinder their interactions with their targets.^95^ For instance, ASOs are single‐stranded oligonucleotides, which form RNA/DNA heteroduplex and induce endogenous RNaseH1‐mediated degradation of targeted lncRNA.^96^ Several designs of ASOs are used in various forms with different mode of actions such as antagonist to NATs (antagoNAT), locked nucleic acid GapmeRs (LNAGapmeRs), and a combination of both to suppress the expression of lncRNAs.^97^, ^98^, ^99^

siRNA is the most commonly used and successful technique to target lncRNA in cancers.^100^ siRNA has already been implicated in the silencing of lncRNAs in many preclinical studies. They are a class of noncoding double‐stranded RNA molecule (19–25 nucleotides) and induce degradation of target transcript followed by base pairing.^100^

Aptamers, single‐stranded nucleic acids (DNA/RNA) with high affinity and specificity to target, can be used against target‐specific lncRNAs sequences.^101^ In addition, they act as nucleic acid analog of antibody, however, with better tissue penetration and transport ability than antibodies. They also generate lower immunogenicity than antibodies.^101^

Furthermore, there are other strategies that have been proposed to interfere with lncRNAs functions including, nanobodies, RNA decoys, and small molecules for disruption of interactions of lncRNA/protein by steric blockade or competitive inhibition.^94^, ^102^ For example, small molecules are promising prospects in the inhibition of binding of either RNA‐binding proteins (RBP) or lncRNA with each other, resulting in alteration of their secondary and/or tertiary‐structures. Small molecular inhibitors can also mask protein‐binding sequences of lncRNAs, thereby prevent the binding of RBPs to the lncRNAs, resulting in disruption of interactions.^101^ However, LncRNA‐protein interactions are not fully revealed, and a clear understanding is required to use this technique in clinical settings.

All of these molecules or techniques could be used to target lncRNAs in TNBC. However, there are challenges regarding the use of aforementioned molecules and techniques and their in vivo applications. Thus, prior to using lncRNAs in TNBC treatment or in clinical trials, lncRNA expression in human must be investigated in animal models in order to identify the interactive networks among lncRNAs, target genes, and their protein products.^103^ Moreover, the main obstacle of using animal models is that among the species lncRNAs are poorly conserved. According, many human lncRNAs are not properly expressed in mice model,^104^, ^105^ only a few lncRNAs (orthologous) were found across mouse and human.^94^ This problem could be overcome by producing humanized mouse‐models, where entire chromosomes or larger human genome segments could be copied.^106^ Therefore, a significant number of researches are imperative to develop effective delivery strategies of these therapeutics to the target sites for the better management of the patients with TNBCs. In addition, most of the preclinical and clinical researches incorporating cellular model in TNBCs were carried out using a single cell line (MDA‐MB‐231), which limit the preclinical validation of the field. Preclinical and clinical studies using of other TNBC cell lines such as HCC1395, HCC1937, MDA‐MB‐436, SUM149PT etc., and development of other functional TNBCs cell lines could provide better insights of the disease, which in turn may help to achieve better clinical outcomes of patients with TNBC.

## 7 CONCLUSIONS AND FUTURE PERSPECTIVE

A complex molecular signaling and extreme physiological/phenotypical heterogeneity is the characteristic feature of cancer patients with TNBC. The poor clinical outcome of patients with TBNC is contributed by this complexity of signaling networks and intrinsic/extrinsic heterogeneity in genetic/epigenetic make up of TBNC. Hence, in this review, we have provided a concise outline regarding the roles of lncRNAs in TNBC pathogenesis, therapy resistance, and prognosis and highlights the importance of lncRNAs in therapy development for TNBC patients. Improved understanding of the roles of lncRNAs in TBNC should promotes new directions for future research and development of therapeutic options for TNBC. Also, the potential therapeutic strategies targeting aberrant activation of lncRNAs were illustrated. However, as discussed, most of the strategies based on targeting and inhibiting mRNA and microRNAs expression. Therefore, a complete understanding is still required to use preclinical knowledge in designing lncRNA targeted therapies in TNBC. In doing so, bioinformatics tools and high‐throughput screening technologies could enrich our knowledge of lncRNAs structure, mechanisms of action, localization, and most importantly of its interrelations with other biological molecules in both cancer cells and normal cells.

## AUTHOR CONTRIBUTIONS

**Plabon Kumar Das:** Conceptualization (equal); data curation (lead); writing – original draft (lead). **Ayesha Siddika:** Data curation (equal); writing – original draft (equal). **K. M. Rashel:** Formal analysis (equal). **Abdul Awal:** Formal analysis (equal). **Kazi Soha:** Formal analysis (equal); resources (equal). **Md Arifur Rahman:** Data curation (equal); resources (equal). **Suja Pillai:** Supervision (equal); writing – review and editing (equal). **Farhadul Islam:** Conceptualization (equal); supervision (lead); writing – review and editing (equal).

## FUNDING INFORMATION

No specific funding was achieved for this project.

## CONFLICT OF INTEREST STATEMENT

There is no conflict of interest among the authors.

## Data Availability

Data sharing is not applicable to this article as no new data were created or analyzed in this study.
